# Efficacy of Eye Movement Desensitization and Reprocessing (EMDR) on Anxiety Severity Among Physicians and Nurses Working in Intensive Care Units

**DOI:** 10.1155/ccrp/7865426

**Published:** 2026-08-08

**Authors:** Nastran Hatami, Mina Jouzi, Sara Kord, Behrooz Zarasvand

**Affiliations:** ^1^ Student Research Committee, Dezful University of Medical Sciences, Dezful, Iran, dums.ac.ir; ^2^ Department of Nursing, Nursing and Midwifery Sciences Development Research Center, Na.C., Islamic Azad University, Najafabad, Iran, azad.ac.ir; ^3^ Department of Nursing, Dezful University of Medical Sciences, Dezful, Iran, dums.ac.ir; ^4^ Department of Neurosurgery, School of Medicine, Dezful University of Medical Sciences, Dezful, Iran, dums.ac.ir

**Keywords:** anxiety, EMDR, healthcare workers, ICU staff, psychological distress, quasi-experimental study

## Abstract

**Background:**

Physicians and nurses working in intensive care units (ICUs) are frequently exposed to emotionally demanding and high‐stress situations, placing them at increased risk for anxiety. Eye movement desensitization and reprocessing (EMDR) has shown promise in reducing anxiety in various high‐stress populations; however, its effectiveness among ICU staff remains underexplored.

**Objective:**

To evaluate the efficacy of EMDR in reducing anxiety severity among ICU physicians and nurses.

**Methods:**

This quasi‐experimental study was conducted among 60 ICU staff members who were allocated to either an EMDR intervention group (*n* = 30) or a control group (*n* = 30). Anxiety severity was assessed using the Beck Anxiety Inventory (BAI) at baseline and post‐intervention. The EMDR protocol consisted of eight sessions delivered twice weekly. Data were analyzed using paired *t*‐tests and ANCOVA controlling for baseline anxiety. Internal consistency of the BAI was assessed using Cronbach’s alpha.

**Results:**

All participants completed the study with no dropouts. Baseline characteristics were comparable between groups. The EMDR group showed a significant reduction in anxiety from baseline to post‐intervention (*p* < 0.01), whereas the control group showed no significant change. ANCOVA revealed a significant effect of EMDR on post‐intervention anxiety scores (F(1,57) = 4.21, *p* = 0.045). Cronbach’s alpha for the BAI in this sample was 0.89.

**Conclusion:**

EMDR significantly reduced anxiety among ICU physicians and nurses. Given the high emotional burden in critical care settings, EMDR may be a valuable component of mental health support programs for healthcare professionals. Further randomized controlled trials with long‐term follow‐up are recommended.

## 1. Background

Physicians and nurses working in intensive care units (ICUs) are routinely exposed to highly stressful clinical environments characterized by rapid patient deterioration, complex decision‐making, high mortality rates, and continuous exposure to traumatic events. Such conditions place ICU staff among the most psychologically vulnerable groups within the healthcare system. Recent evidence indicates that the prevalence of anxiety among ICU personnel is considerably high, with studies reporting rates ranging from 41% to 70% depending on workload, staffing shortages, and exposure to critical incidents [1, 2]. Anxiety in this population is strongly associated with impaired clinical performance, increased medical errors, burnout, and reduced quality of patient care [3, 4]

Given the substantial psychological burden experienced by ICU personnel, identifying effective, nonpharmacological interventions to reduce anxiety has become a priority in occupational mental health research. One promising approach is eye movement desensitization and reprocessing (EMDR), a structured psychotherapy developed by Shapiro and grounded in the Adaptive Information Processing (AIP) model. According to the AIP framework, unprocessed distressing memories remain stored in a maladaptive form, contributing to emotional dysregulation and anxiety.

EMDR facilitates the reprocessing of distressing memories through bilateral stimulation—typically eye movements—leading to reduced emotional reactivity and the integration of more adaptive cognitions [5, 6] EMDR has demonstrated efficacy across a wide range of psychological conditions, including post‐traumatic stress disorder (PTSD) [7], stress and anxiety in emergency medical technicians [5], anxiety in myocardial infarction patients [8, 9], suicidal ideation in major depressive disorder [10], and pandemic‐related PTSD in frontline healthcare workers [10]. These findings suggest that EMDR may be particularly suitable for healthcare professionals who are repeatedly exposed to emotionally challenging clinical situations.

Despite the expanding body of EMDR research, its application specifically for reducing anxiety among ICU physicians and nurses remains insufficiently explored. While several studies have examined anxiety, burnout, and occupational stress in ICU staff, very few have evaluated targeted psychotherapeutic interventions designed to mitigate these symptoms. Existing EMDR studies have predominantly focused on clinical populations or first responders, leaving a critical gap regarding its effectiveness in nonclinical but high‐risk occupational groups such as ICU personnel.

Considering the high prevalence of anxiety among ICU staff and the promising outcomes of EMDR in related populations, investigating EMDR as a feasible and effective intervention for this group is both timely and necessary. Therefore, the present study aimed to evaluate the efficacy of EMDR therapy in reducing anxiety severity among physicians and nurses working in the ICUs of Ganjavian Hospital in Dezful. This study addresses an important gap in the literature and contributes to the development of evidence‐based mental health strategies for critical care professionals.

## 2. Objective

To examine the effect of EMDR therapy on the severity of anxiety experienced by physicians and nurses employed in the ICUs of Ganjavian Hospital in Dezful.

## 3. Methods

### 3.1. Study Design and Setting

**FIGURE 1:**
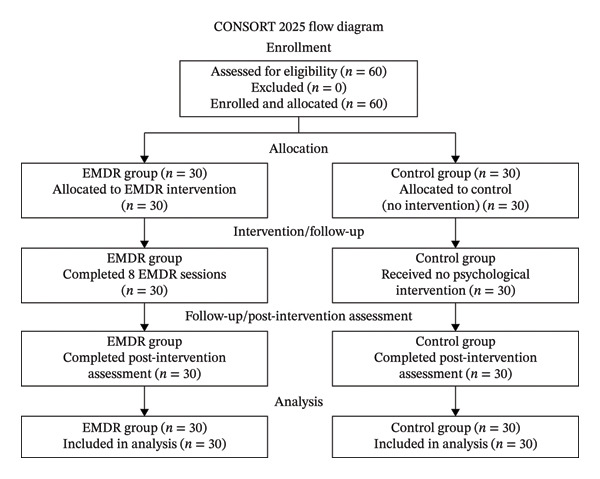
The CONSORT diagram for study selection.

This study employed a quasi‐experimental, parallel‐group design conducted in 2024 in the ICUs of Ganjavian Hospital, Dezful, Iran. The study followed the CONSORT 2025 extension for non‐randomized trials, ensuring transparent reporting of participant flow, intervention procedures, and outcome assessment. Figure 1 shows The CONSORT diagram for study selection.

### 3.2. Participants and Sampling

Participants were recruited through convenience sampling, which may limit external validity; therefore, the potential impact of this sampling method on generalizability is acknowledged. Eligible participants included ICU physicians and nurses who met the following criteria:

• age < 70 years
• no history of seizures
• no visual or hearing impairment
• no substance use disorder
• willingness to participate and provide written informed consent

#### 3.2.1. Exclusion Criteria Included

• unwillingness to continue participation
• intolerance to treatment, defined as emotional overwhelm, inability to complete bilateral stimulation, or clinician‐determined risk of psychological destabilization
• absence from more than one EMDR session
• incomplete questionnaire data

#### 3.2.2. Group Assignment

Although the study used convenience sampling, participants were assigned to intervention or control groups using a computer‐generated allocation list to minimize selection bias. Allocation was performed by an independent researcher not involved in data collection or intervention delivery.

### 3.3. Outcome Measure

#### 3.3.1. Beck Anxiety Inventory (BAI)

Anxiety severity was assessed using the 21‐item BAI, scored on a 0–63 scale. The BAI demonstrates strong psychometric properties, including:

• Cronbach’s alpha in the present study: 0.89
• previously reported reliability: α = 0.92
• test–retest reliability: *r* = 0.75
• validated Persian version with strong construct validity

#### 3.3.2. Interpretation of Scores

• 0–7: minimal anxiety
• 8–15: mild anxiety
• 16–25: moderate anxiety
• 26–63: severe anxiety

### 3.4. Timing of BAI Administration

To address reviewer concerns, the BAI was administered at two time points:

1. Baseline (pre‐intervention)
2. Immediately after completion of the 8 EMDR sessions (post‐intervention)

No mid‐treatment or follow‐up assessments were conducted.

## 4. EMDR Intervention

The intervention followed Shapiro’s standard eight‐phase EMDR protocol, delivered in eight sessions, each lasting 45–60 min. Sessions were conducted twice weekly (not biweekly)—meaning two sessions per week, not once every 2 weeks.

### 4.1. Rationale for Eight Sessions

Although ICU staff are a nonclinical population, they are repeatedly exposed to traumatic and emotionally distressing events. Previous EMDR studies in emergency medical technicians, myocardial infarction patients, and frontline healthcare workers have successfully used 6–8 sessions, supporting the appropriateness of this dosage.

### 4.2. Therapist Qualifications

All EMDR sessions were administered by a licensed clinical psychologist with EMDR Europe–approved training, holding certification in EMDR Level II.

### 4.3. Target Selection

EMDR targets were identified during Phase 1 and Phase 3 through:

• exploration of recent distressing ICU‐related events
• memories associated with patient death, ethical dilemmas, or perceived failure
• current triggers contributing to anxiety

Targets were selected collaboratively based on the participant’s subjective units of disturbance (SUD) and negative cognition strength.

#### 4.3.1. Handling Early Termination

If a participant completed processing before eight sessions:

• remaining sessions were used for installation of positive cognition,
• future template work,
• and stabilization techniques.

If a participant could not continue due to intolerance or scheduling issues, they were withdrawn according to exclusion criteria.

#### 4.3.2. Control Group

Participants in the control group received no psychological intervention during the study period but completed all assessments. After study completion, they were offered EMDR sessions as an ethical consideration.

### 4.4. Data Analysis

Data were analyzed using SPSS v21. Descriptive statistics summarized demographic variables. Between‐group comparisons were conducted using:

• independent t‐tests (continuous variables)
• chi‐square tests (categorical variables)
• ANCOVA to compare post‐intervention anxiety while adjusting for baseline scores

Within‐group comparisons (requested by reviewers) were performed using paired *t*‐tests.

Statistical significance was set at *p* < 0.05. These baseline comparisons are detailed in Table 1.

**TABLE 1:** Baseline demographic and clinical characteristics of participants.

Variable	EMDR group (*n* = 30)	Control group (*n* = 30)	Statistical test	*p* value
Age (years), mean ± SD	32.40 ± 3.27	34.30 ± 3.23	Independent t‐test	0.634
Profession			Chi‐square	0.597
Physician, *n* (%)	11 (36.7%)	13 (43.3%)		
Nurse, *n* (%)	19 (63.3%)	17 (56.7%)		
Marital status			Chi‐square	0.700
Single, *n* (%)	12 (40.0%)	13 (43.3%)		
Married, *n* (%)	18 (60.0%)	17 (56.7%)		
Baseline anxiety (BAI), mean ± SD	18.90 ± 10.93	19.50 ± 12.84	Independent t‐test	0.846

## 5. Results

Participant Flow: A total of 60 ICU physicians and nurses were screened for eligibility, all of whom met the inclusion criteria and were enrolled in the study. Participants were then allocated to the EMDR group (*n* = 30) or the control group (*n* = 30). No dropouts occurred during the intervention or follow‐up period, and all participants were included in the final analysis.

Baseline Characteristics: Baseline demographic and clinical characteristics were comparable between the two groups (Table 1). The mean age did not differ significantly between the EMDR group (32.40 ± 3.27 years) and the control group (34.30 ± 3.23 years) (t(58) = −0.48, *p* = 0.634). The distribution of profession (physician vs. nurse) and marital status was also statistically similar between groups (*p* > 0.05). Baseline anxiety scores measured by BAI showed no significant difference between the EMDR (18.90 ± 10.93) and control groups (19.50 ± 12.84) (t(58) = −0.19, *p* = 0.846). Table 1 shows baseline demographic and clinical characteristics of participants.

### 5.1. Effect of EMDR on Anxiety Severity

Following the intervention, the EMDR group demonstrated a significant reduction in anxiety severity, with mean post‐intervention BAI scores decreasing to 14.46 ± 10.12. In contrast, the control group showed a minimal, nonsignificant reduction (18.56 ± 12.24).

To adjust for baseline differences, an ANCOVA was conducted with baseline BAI scores entered as a covariate. The analysis revealed a significant main effect of the EMDR intervention on post‐intervention anxiety levels:

F(1, 57) = 4.21, *p* = 0.045, partial η^2^ = 0.069

indicating that EMDR produced a statistically meaningful reduction in anxiety compared with the control condition. Table 2 shows post‐intervention anxiety scores.

**TABLE 2:** Post‐intervention anxiety scores (ANCOVA adjusted for baseline).

Group	Mean BAI score (post‐intervention)	SD	ANCOVA result
EMDR	14.46	10.12	F (1, 57) = 4.21, *p* = 0.045, partial *η* ^2^ = 0.069
Control	18.56	12.24	—

## 6. Discussion

The present quasi‐experimental study examined the efficacy of EMDR in reducing anxiety severity among physicians and nurses working in ICUs. The findings demonstrated that EMDR produced a statistically significant reduction in anxiety compared with the control group, indicating that EMDR can be an effective intervention for occupational anxiety among healthcare professionals exposed to high‐stress environments. These results are consistent with previous studies showing that EMDR is effective in reducing anxiety symptoms in both clinical and nonclinical populations exposed to stressful or traumatic events [11, 12].

Several studies have reported that healthcare workers, particularly those working in emergency and critical care settings, experience high levels of anxiety, burnout, and psychological distress due to repeated exposure to patient suffering, ethical dilemmas, and life‐threatening situations [12–15].

The significant reduction in anxiety observed in the present study aligns with findings from research conducted among emergency medical technicians, frontline healthcare workers, and disaster responders, where EMDR demonstrated moderate to large effect sizes in reducing anxiety and stress‐related symptoms [5, 16, 17].

Our results extend this evidence to ICU physicians and nurses, a population that has been relatively understudied in EMDR research [18, 19].

The mechanism through which EMDR reduces anxiety can be understood through the AIP model proposed by Shapiro [20, 21].

According to this model, distressing experiences may be stored in a maladaptive form, leading to persistent emotional reactivity and dysfunctional beliefs. EMDR facilitates the reprocessing of these maladaptively stored memories through bilateral stimulation, allowing them to be integrated into more adaptive memory networks. Neurobiological studies suggest that EMDR may modulate limbic system activation, enhance prefrontal regulatory control, and promote memory reconsolidation, thereby reducing the emotional intensity of previously distressing experiences.

These mechanisms may be particularly relevant for ICU staff, who frequently encounter emotionally charged clinical events such as patient deterioration, resuscitation failures, and end‐of‐life care.

The magnitude of anxiety reduction observed in this study is comparable to findings from previous EMDR trials conducted among healthcare workers. For example, studies among emergency nurses and paramedics have shown that EMDR significantly reduces anxiety, intrusive thoughts, and physiological arousal.

Similarly, research conducted during the COVID‐19 pandemic demonstrated that EMDR effectively reduced anxiety and distress among frontline healthcare workers.

The consistency of these findings across different high‐stress medical populations supports the robustness of EMDR as a brief and efficient intervention.

An important implication of the present study is that EMDR can be delivered in a relatively short format—eight sessions—while still producing clinically meaningful reductions in anxiety. This is particularly valuable in ICU settings, where staff often have limited time and high workloads. Integrating EMDR into staff support programs may enhance psychological resilience, reduce occupational stress, and potentially improve patient care outcomes.

Despite these promising findings, several limitations should be acknowledged. First, the use of convenience sampling may limit the generalizability of the results, as participants who volunteered may differ from those who did not. This potential selection bias should be considered when interpreting the findings. Second, although random allocation was used, the quasi‐experimental design does not provide the same level of causal inference as a fully randomized controlled trial. Third, the study relied solely on self‐report measures, which may be subject to response bias. Incorporating clinician‐rated or physiological measures could strengthen future research. Fourth, the absence of long‐term follow‐up assessments prevents conclusions about the durability of EMDR’s effects. Future studies should include follow‐up evaluations to determine whether reductions in anxiety are maintained over time.

In conclusion, the findings of this study indicate that EMDR is an effective intervention for reducing anxiety among ICU physicians and nurses. Given the high emotional demands of critical care environments, EMDR may serve as a valuable component of mental health support programs aimed at enhancing resilience and reducing psychological distress among healthcare professionals. Future research should employ larger randomized controlled trials, incorporate long‐term follow‐up, and explore the integration of EMDR into comprehensive occupational mental health frameworks.

## 7. Conclusion

The findings of this quasi‐experimental study demonstrate that EMDR is an effective intervention for reducing anxiety severity among physicians and nurses working in ICUs. Given the high emotional demands and continuous exposure to stressful clinical situations in critical care settings, EMDR offers a brief, structured, and clinically meaningful approach to enhancing psychological well‐being among healthcare professionals. Although the study’s quasi‐experimental design and convenience sampling limit the generalizability of the findings, the significant reduction in anxiety observed in the intervention group highlights the potential value of integrating EMDR into staff support and mental health programs. Future research should employ larger randomized controlled trials, incorporate long‐term follow‐up assessments, and explore the comparative effectiveness of EMDR relative to other evidence‐based interventions. Overall, EMDR may serve as a valuable component of comprehensive strategies aimed at improving resilience and reducing psychological distress among ICU staff.

## Author Contributions

Sara Kord contributed to the study design, data collection, statistical analysis, and manuscript preparation. Nastran Hatami, Behrooz Zarasvand, and Mina Jouzi assisted with data collection, literature review, and manuscript drafting. Sara Kord provided guidance on the study design, statistical analysis, and manuscript review. All the authors have read and approved the final manuscript.

## Funding

This research received no specific grant from funding agencies in the public, commercial, or not‐for‐profit sectors. The study was supported by institutional resources at Dezful University of Medical Sciences.

## Disclosure

A preprint of this work has previously been published [Sara Kord Nastran Hatami, Behrooz Zarasvand, Mina Jouzi Impact of Eye Movement Desensitization and Reprocessing (EMDR) on the Severity of Anxiety among Physicians and Nurses Working in Intensive Care Units].

## Ethics Statement

This study received full ethical approval from the Ethics Committee of Dezful University of Medical Sciences, Dezful, Iran (Approval Number: IR.DUMS.REC.1401.033). This research was conducted in strict accordance with the ethical principles outlined in the Declaration of Helsinki, ensuring the protection of participants’ rights and well‐being.

## Consent

All participants provided informed consent prior to their enrollment, confirming their voluntary participation after a comprehensive explanation of the study’s objectives, procedures, potential risks, and benefits. Furthermore, participants granted explicit consent for the publication of their anonymized data, maintaining their privacy and confidentiality throughout the dissemination process.

## Conflicts of Interest

The authors declare no conflicts of interest.

## Data Availability

The data supporting the findings of this study are available from the corresponding author upon reasonable request.
